# Suicide warning signs of self-identification in patients with mood disorders: a qualitative analysis based on safety planning

**DOI:** 10.3389/fpubh.2024.1417817

**Published:** 2024-08-21

**Authors:** Honghui Zhang, Jiaxin Zhu, Lu Niu, Meng Zeng, Tengwei Chen, Yuedong Chen, Xiaofei Hou, Haojuan Tao, Yarong Ma, Kangguang Lin, Liang Zhou

**Affiliations:** ^1^Department of Social Medicine and Health Management, Xiangya School of Public Health, Central South University, Changsha, China; ^2^Tianjin Anding Hospital, Mental Health Center of Tianjin Medical University, Tianjin, China; ^3^National Clinical Research Center for Mental Disorders, and Department of Psychiatry, The Second Xiangya Hospital of Central South University, Changsha, China; ^4^The Affiliated Brain Hospital of Guangzhou Medical University, Guangzhou, China; ^5^School of Health and Life Sciences University of Health and Rehabilitation Sciences, Qingdao, China

**Keywords:** suicide, warning signs, mood disorders, safety planning, network analysis, content analysis, China

## Abstract

**Introduction:**

Warning signs serve as proximal indicators of suicide risk, making early recognition imperative for effective prevention strategies. This study aimed to explore self-identified suicide warning signs among Chinese patients with mood disorders based on safety planning framework.

**Methods:**

Researchers collaborated with patients to develop a safety plan and compiled warning signs based on it. Word frequency and network analysis were conducted to identify key warning signs. Directed content analysis categorized these signs into cognitive, emotional, behavioral, or physiological themes according to the suicide mode theory. Additionally, we examined potential variations in reported warning signs among participants with different demographic characteristics, including age, gender, and history of suicide attempts.

**Results:**

“Low mood” and “crying” emerged as prominent warning signs, with “social withdrawal” closely following. Patients commonly reported emotional themes during suicidal crises, often experiencing two to three themes simultaneously, primarily focusing on emotional, behavioral, and physiological themes. Males exhibited a higher proportion of concurrently reporting three sign themes compared to females (*P* < 0.05), while no difference was observed in warning signs among patients with other demographic traits.

**Discussion:**

This study offers a nuanced understanding of warning signs among mood disorder patients in China. The findings underscore the necessity for comprehensive suicide risk management strategies, emphasizing interventions targeting emotional regulation and social support. These insights provide valuable information for enhancing suicide prevention and intervention efforts.

## 1 Introduction

Suicide is a significant global public health issue, and its prevalence among young people has been particularly concerning. The World Health Organization (WHO) estimates that suicide is the second leading cause of death among individuals aged 15–29 globally (1). In China, suicide remains a major cause of mortality among young people (2), with recent studies reported that suicide rates among people aged 10–24 years was 2.86 per 100,000 individuals in 2021 (3) and with an average annual growth rate of 17.17% from 2017 to 2021 (4). Furthermore, adolescents diagnosed with mood disorders are at heightened risk of suicide (5). A meta-analysis has indicated that the suicide rate among individuals with mood disorders exceeds that of the general adolescent population by more than 30 times (6). Thus, research on suicide within this demographic holds significant public health implications.

Current suicide prevention practices in China involve a multi-faceted approach, including mental health education, crisis intervention hotlines, psychological counseling services, medication, and hospitalization (7). In addition to these efforts, brief social psychological interventions, such as safety planning, have gained more and more attention due to their simplicity, effectiveness, and cost-efficiency (8). Safety plans have emerged as a promising intervention to mitigate the risk of suicide among high-risk groups (9). A safety plan is a personalized, practical plan that includes strategies and resources to help individuals cope during a crisis. It typically involves identifying warning signs, employing coping strategies, contacting support networks, and accessing emergency services (10).

Warning signs are defined as “the earliest detectable signs indicating increased risk of suicide in the near-term (i.e., within minutes, hours, or days),” highlighting their proximal relationship to suicidal behavior (11). However, compared to the extensive literature on distal risk factors, there is limited data on warning signs (12). While the presence of risk factors may indicate the possibility of engaging in suicide-related behaviors at some point throughout one's life (i.e., “chronic” factors), it does not provide information about an individual's imminent risk for suicide attempt (13). Therefore, the timely and effective identification of suicide warning signs holds greater potential for saving lives among high-risk individuals.

The identification of warning signs for acute suicide risk has been a central focus for researchers, clinicians, and other stakeholders in the field of suicide prevention for decades. In 2003, experts proposed a consensus list containing 12 warning signs, which has since been widely cited in scientific literature and extensively disseminated to the public (11). Currently, empirical research on warning signs has made some new advancements. Scholars have reported specific warning signs among various at-risk populations, including gun owners (14), active-duty soldiers (15), and adult patients hospitalized for suicide attempts (16). Gender differences in warning signs have also been investigated, with agitation being associated with suicide attempts in male psychiatric inpatients but not in females (17). Furthermore, some researchers have employed controlled study designs to validate warning signs that significantly influence suicidal behavior, thereby providing valuable insights for acute risk management decisions (18). However, there remains a significant gap in empirical research on suicide warning signs within the specific cultural context of China. Given the unique socio-cultural factors that may influence the manifestation and interpretation of warning signs among Chinese populations, it is imperative to conduct targeted research in this area.

Hence, this study aimed to achieve three primary objectives: Firstly, to compile warning signs from participant safety plans in a sample of hospital outpatients; Secondly, to employ word frequency analysis and network analysis to elucidate the characteristics of these warning signs; Thirdly, to encode and categorize warning signs guide by suicide mode, and to examine potential variations in reported warning signs among participants with different demographic characteristics. Through these objectives, our study sought to deepen our understanding of suicide warning signs among Chinese patients diagnosed with mood disorders.

## 2 Materials and methods

### 2.1 Participants and recruitment

Participants were recruited from outpatient psychiatric clinics in three regions of China between November 2021 and December 2023 using convenience sampling (19). All participants fulfilled the following inclusion criteria: (1) age from 12 to 25; (2) self-reported suicidal ideation within the past 2 weeks; and (3) diagnoses met the criteria for mood disorder as defined by the International Classification of Diseases (ICD-10). Exclusion criteria were: (1) previous or current psychotic symptoms or other psychiatric illness; (2) being judged by a psychiatrist to be currently having a manic episode; (3) inability to provide informed consent or answer self-assessment questions due to cognitive impairment.

Approvals were obtained from the Institutional Review Boards (IRBs) of the Xiangya School of Public Health, Central South University (XYGW-2021-73 and XYGW-2022-39), the Affiliated Brain Hospital of Guangzhou Medical University (2021–089) and Tianjin Anding Hospital (2023–01 and 2023–02). Written informed consent was obtained from all participants (and one of their parents if they were under 18 years old). This study is one part of a larger project (20) (Zhu et al., manuscript submitted)^1^ and participants received a smartband (~$25 US dollar) as compensation for their involvement in the baseline assessments, which included the safety plan intervention.

### 2.2 Data sources

After obtaining informed consent, researchers instructed patients to complete a series of self-report questionnaires to collect personal information, including demographic data (e.g., age, gender, ethnicity, highest level of education, etc.) and clinical data (suicidal ideation, depression levels and the history of past suicide attempts). Suicidal ideation within a week was assessed using the Chinese Version of the Beck Scale for Suicide Ideation (BSI-CV) (21). In this study, the BSI-CV demonstrated good internal consistency (Cronbach's α = 0.84, 95% CI : 0.790–0.878). Depression levels were measured using the Patient Health Questionnaire-9 (PHQ-9), where scores of 5, 10, 15, and 20 indicate mild, moderate, moderately severe, and severe depression, respectively (22). The PHQ-9 also showed good internal consistency in the current study (Cronbach's α = 0.76, 95% CI: 0.687–0.816). The history of past suicide attempts was investigated with the question: “Have you ever attempted suicide in your life?”

Researchers then collaborated with patients to develop a safety plan, a process that typically lasted between 20 and 40 min. Initially, researchers explained the purpose of the safety plan and its role in managing suicide risk. Following this, patients were guided to describe their most recent suicide crisis, recounting events before, during, and after the crisis, including triggers and their responses (10). A suicide crisis could involve the most recent suicide attempt or, if there had been no recent suicidal behavior, a recent period of intense or severe suicidal thoughts (10, 23). This narrative helped identify individual warning signs and outline the steps of the safety plan. Finally, researchers transcribed this information onto two paper forms: one copy was given to the patient, and the other was retained by the research team. Warning signs extracted from the safety plans were analyzed for subsequent analysis.

### 2.3 Quality control

To ensure the objectivity of the research process and the reliability of the data, we implemented the following quality control measures: First, all investigators underwent rigorous training prior to the development of the safety plan, including studying Stanley & Brown's manual on safety plans (24), watching official videos (Suicide Prevention | The Joint Commission), and participating in role-playing exercises guided by clinical psychiatrists. During the creation of the safety plan, with patient consent, we recorded the process, which was later reviewed by clinicians and crisis intervention experts to ensure quality. Secondly, during the process of identifying warning signs, we extensively engaged with participants to encourage genuine expression. Drawing from the Safety Plan Treatment Manual (24), we used prompts such as “How will you know when to use the safety plan?” and “What do you experience when you start to think about suicide or feel extremely depressed?” to elicit responses in the participants' own words. Additionally, we minimized researcher intervention and maintained neutrality whenever possible.

### 2.4 Data analysis

#### 2.4.1 Word frequency analysis

The text containing warning signs was copied into a document, and the ROST ContentMining software was utilized for word frequency analysis. Characteristic terms such as “passive suicidal ideation” and “social withdrawal” were incorporated into a customized dictionary. Subsequently, the document underwent segmentation and word frequency calculation using the software. Following filtering, meaningful high-frequency characteristic words along with their frequencies were obtained. To present the word frequency analysis results in the paper, we translated high-frequency Chinese words into English. These translated English terms were then visualized using a word cloud (https://www.wordclouds.com/), where darker colors and larger characters signify higher probabilities (25).

#### 2.4.2 Network analysis

High-frequency words merely reflect the primary domains of concepts through extracted phrase attributes, while co-occurrence network analysis utilizes network structures (nodes and edges) to quantify diverse relationships between words, thereby uncovering inherent associations among high-frequency terms (26).

In this study, we generated an initial keyword co-occurrence matrix using ROST and visualized the network structure with Gephi software (27). Within Gephi, we employed the Fruchterman Reingold layout to depict intricate networks, effectively illustrating spatial relationships and connection densities among nodes. During visualization, nodes represented warning signals within the text, with node size correlating to their degree, indicating their significance; edges portrayed co-occurrence links between nodes, with thicker edges denoting stronger connections, reflecting the proximity and frequency of co-occurrence among key terms. Additionally, we utilized Gephi's statistical analysis module to compute topological parameters such as degree centrality, closeness centrality, and betweenness centrality, facilitating a deeper analysis of the network's complexity and the significance of pivotal nodes (28). Furthermore, we conducted comparative analysis of network structures based on gender (male or female).

#### 2.4.3 Content analysis

Directed qualitative content analysis is particularly well-suited for scenarios where prior knowledge of the subject is available. This method involves categorizing data using a set of predefined codes (deductive coders) created from existing theories or frameworks (29–31). In this study, we utilized the Suicide Mode framework proposed by Rudd as the coding framework (32). The Suicide Mode conceptualizes a network of cognitive, behavioral, emotional, and physical experiences that are simultaneously activated in response to triggering events, such as life stressors. These internal experiences interact with external life events, contributing to the maintenance of suicidal thoughts and behaviors (33). Guided by the Suicide Mode framework (32) and referencing existing literature (11, 14, 16, 34) and incorporating our newly summarized findings from a qualitative review of transcripts, we developed a coding system aligned with warning signs (Supplementary material). This system comprises 22 subcategories (considered deductive coding) and 4 main categories (cognitive, emotional, behavioral, physiological), enabling a systematic understanding and classification of the various signs exhibited by patients.

The coding process was independently conducted by two coders across three stages. Initially, warning signs were extracted from safety plans, such as “can't control my tears”. Then, both coders independently conducted primary coding on the extracted warning signs according to a predefined coding framework (Supplementary material). For instance, “can't control my tears” was categorized as “emotional pain (sadness, emptiness, hurt)”. Finally, these preliminary themes were compared with the four established structures of the suicide model and independently categorized by the two coders into behavioral, cognitive, emotional, and physical themes. For example, “emotional pain (sadness, emptiness, hurt)” was categorized under “emotional”. More coding examples can be found in Table 1. Coding consistency was evaluated using kappa values, with subcategory coding achieving a kappa of 0.80 and main category coding achieving 0.82, indicating strong consistency in coding outcomes (35). Any discrepancies encountered during coding were resolved through conference discussions, and the final coding scheme was endorsed by senior researchers.

**Table 1:** Content, code, and categories of warning signs from participant safety planning.

**Example warning signs**	**Code**	**Category**
I felt hopeless	Hopelessness	Cognitive
Feelings of chest tightness and breathlessness	Chest issues	Physiological
I just want to be in my room by myself	Social withdrawal	Behavioral
Poor appetite	Stomach issues	Physiological
Unable to concentrate	Cognitive disturbance	Cognitive
Make a suicide plan, buy tools	Making preparations of personal affairs to attempt suicide	Behavioral
A very strong sense of anxiety	Anxiety/tension/fear	Emotional
I couldn't stop crying	Emotional pain (sadness, emptiness, hurt)	Emotional
I lose my temper easily	Irritability	Physiological

Finally, a series of chi-square analyses were performed to examine differences in reported warning signs among different demographic groups (e.g., age, gender, history of suicide attempts). All statistical analyses were conducted using SPSS 26.0.

## 3 Results

As shown in Table 2, of the 124 participants, the majority were females (71.8%) and most were high school or university students (78.2%), with a mean age of 19.2 ± 3.0. Among these participants, 74.2% were diagnosed with depression and 25.8% with bipolar disorder. The average scores for depression and suicidal ideation at baseline were 18.8 ± 4.9 and 14.7 ± 6.8, respectively. Additionally, 47.6% reported a history of suicide attempts.

**Table 2:** Sociodemographic and clinical characteristics of participants (*n* = 124).

**Variable**	***M* ±SD (*n*/%)**
**Gender**	
Male	35 (28.2)
Female	89 (71.8)
**Age** ^a^	19.2 ± 3.0
Young adult	79 (63.7)
Adolescent	45 (36.3)
**Residence**	
Urban	85 (68.5)
Rural	39 (31.5)
**Educational**	
Junior high school	22 (17.7)
High school/secondary school/higher vocational school	41 (33.1)
Junior college/university	61 (49.2)
**Occupation status**	
Student	97 (78.2)
Working	19 (15.3)
Unemployed	8 (6.5%)
**Diagnose**	
Depressive disorder	92 (74.2)
Bipolar disorder	32 (25.8)
**Depression (PHQ-9)** ^b^	18.8 ± 4.9
Mild	8 (6.5)
Moderate	16 (12.9)
Moderately severe	43 (34.7)
Severe	57 (45.9)
**Suicidal ideation within a week (BSI-CV)** ^c^	14.7 ± 6.8
**Suicide attempt in the past year**	
Yes	59 (47.6)
No	65 (52.4)

^a^Age: classified based on being under or over 18 years old.

^b^The PHQ-9 was rated on a scale of 0–27.

^c^The BSI-CV was rated on a scale of 0–38.

### 3.1 Word frequency analysis

**Figure 1:**
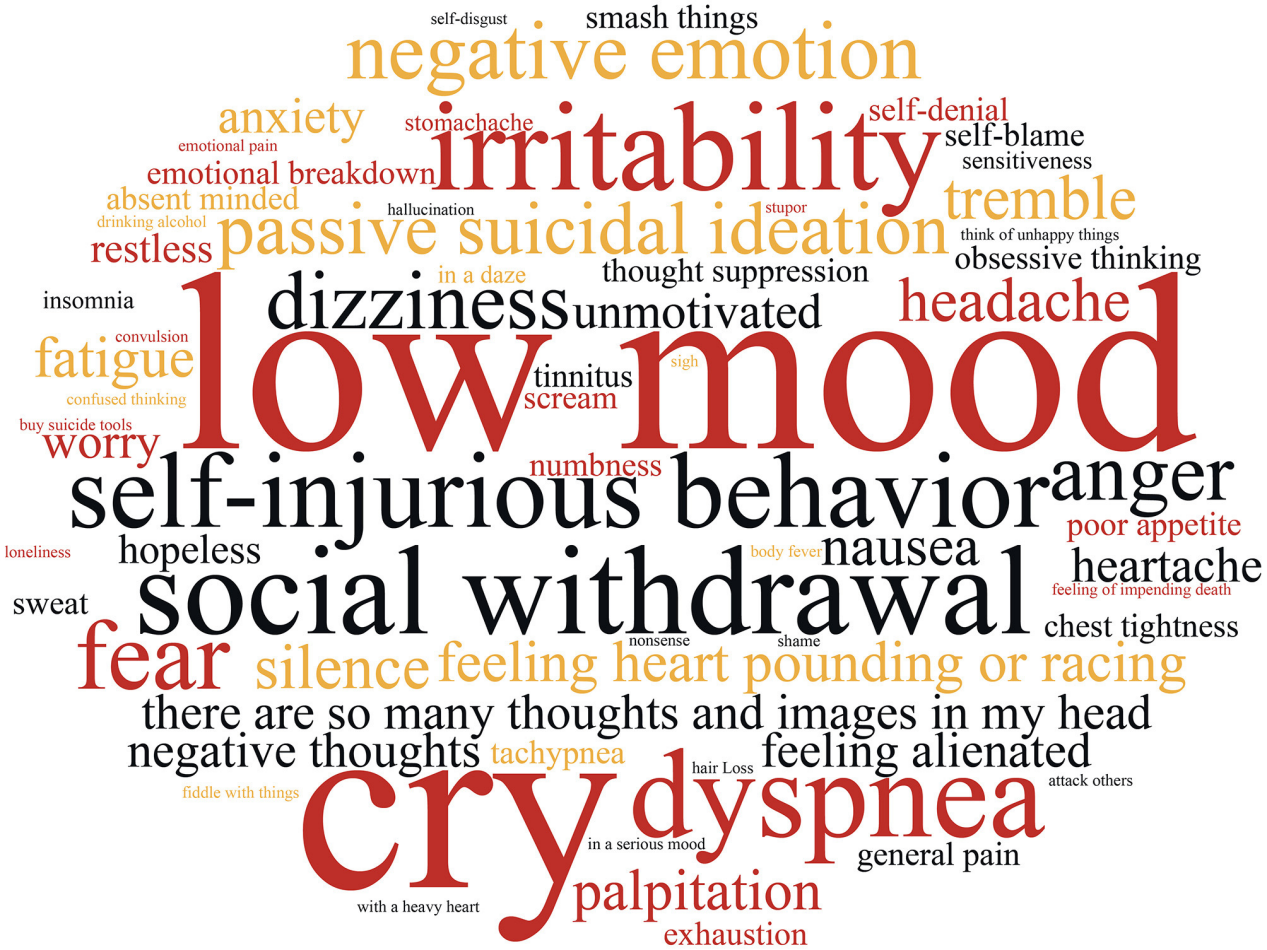
Word cloud visualization of warning signs.

We compiled a total of 442 warning sign items from 124 safety plans. After processing with the ROST software, including tokenization and synonym merging, a total of 69 keywords were generated, as illustrated in Figure 1. The size of each word in the image corresponds to its frequency, with “low mood” emerging as the most common keyword, followed by “cry,” “social withdrawal,” “irritability,” and “tremble.” To provide a more direct visualization of the distribution of high-frequency keywords, the top 30 words were extracted and are presented in Table 3.

**Table 3:** The frequency, degree, closeness centrality, and betweenness centrality of the top 30 keywords.

**Rank**	**Word**	**Frequency (number)**	**Word**	**Degree**	**Word**	**Closeness centrality**	**Word**	**Betweenness centrality**
1	Low mood	46	Low mood	21	Low mood	0.717391304	Low mood	1
2	Cry	37	Cry	18	Cry	0.673469388	Social withdrawal	0.45451977
3	Social withdrawal	30	Social withdrawal	15	Social withdrawal	0.634615385	Shiver	0.415560622
4	Irritability	24	Irritability	14	Irritability	0.622641509	Cry	0.384408082
5	Tremble	17	Shiver	14	Headache	0.611111111	Headache	0.259500177
6	Silence	16	Headache	13	Shiver	0.611111111	Irritability	0.209333157
7	Dyspnea	16	Dizziness	12	Dyspnea	0.578947368	Dizziness	0.202291561
8	Headache	15	Dyspnea	11	Dizziness	0.578947368	Exhaustion	0.176474551
9	Self-injurious behavior	13	Silence	10	Silence	0.568965517	Unmotivated	0.175257485
10	Unmotivated	12	Feeling heart pounding or racing	8	Feeling heart pounding or racing	0.540983607	Silence	0.085642317
11	Dizziness	10	Non-suicidal self-injury	7	Non-suicidal self-injury	0.523809524	Dyspnea	0.047411236
12	Negative emotion	10	Nausea	5	Nausea	0.515625	Worry	0.031400299
13	Passive suicidal ideation	9	Palpitation	5	Physical fatigue	0.507692308	Non-suicidal self-injury	0.01591531
14	Palpitation	7	Physical fatigue	5	Palpitation	0.492537313	Restlessness	0.007028555
15	Fatigue	7	Unmotivated	5	Heartache	0.47826087	Feeling heart pounding or racing	0.005324663
16	Feeling heart pounding or racing	7	Worry	5	Unmotivated	0.471428571	Anxiety	0.005111677
17	chest tightness	7	Exhaustion	4	Passive suicidal ideation	0.458333333	Nausea	0.001369199
18	Nausea	7	Anxiety	3	Tenseness	0.458333333	Physical fatigue	0.001217066
19	Heartache	6	Chest tightness	3	Chest tightness	0.452054795	Palpitation	0
20	Anxiety	6	Heartache	3	Worry	0.445945946	Heartache	0
21	Worry	6	Restlessness	3	Anxiety	0.44	Passive suicidal ideation	0
22	Negative thoughts	6	Passive suicidal ideation	2	Exhaustion	0.44	Tenseness	0
23	Chest tightness	6	Smash things	2	Restlessness	0.428571429	Chest tightness	0
24	Fear	6	Stomachache	2	Anger	0.423076923	Anger	0
25	There are so many thoughts and images in my head	6	Tenseness	2	Body fever	0.423076923	Body fever	0
26	Restless	5	There are a lot of thoughts in my head	2	Insomnia	0.423076923	Insomnia	0
27	Hopeless	5	Anger	1	Negative emotion	0.423076923	Negative emotion	0
28	Anger	5	Anxiety	1	Smash things	0.4125	Smash things	0
29	Scream	4	Body fever	1	Stomachache	0.402439024	Stomachache	0
30	Thought suppression	4	Insomnia	1	There are a lot of thoughts in my head	0.402439024	There are a lot of thoughts in my head	0

### 3.2 Network analysis

**Figure 2:**
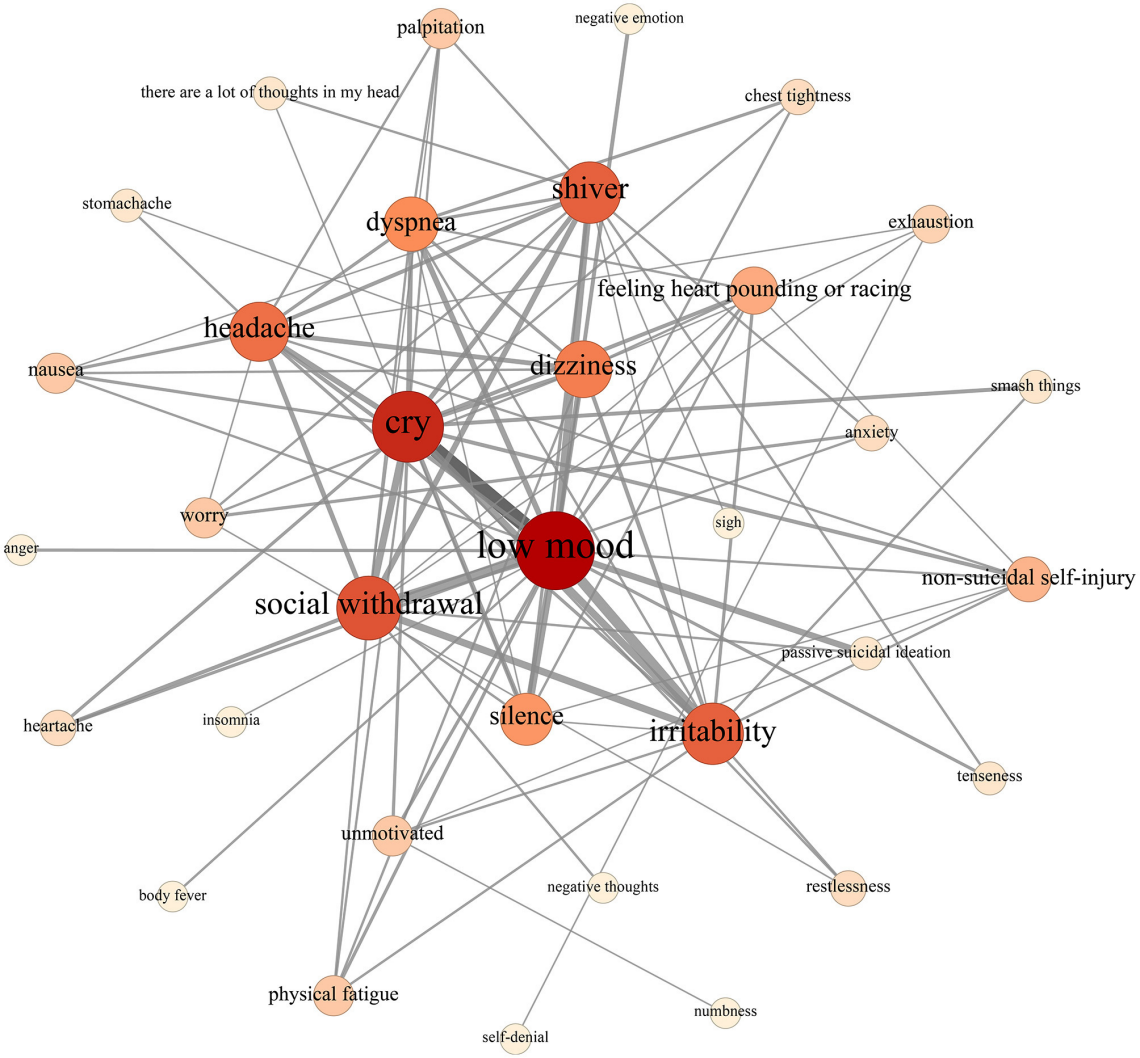
Network structure visualization of warning signs.

The network structure of warning signs is illustrated in Figure 2. Notably, nodes representing “low mood” and “cry” are the most prominent, with “tremble,” “irritability,” “social withdrawal,” and “dyspnea” following closely behind, underscoring their central importance in the network. Examination of the line thickness reveals that words closely associated with “low mood” include “cry,” “social withdrawal,” “irritability,” and “silence,” while words closely linked to “cry” are “irritability” and “social withdrawal”. Detailed information regarding the degree centrality, closeness centrality, and betweenness centrality of each node is provided in Table 3.

This network has 34 nodes (warning sign words) and 101 communication links between nodes. The density of this network is 0.18, meaning that out of 100% possible connections between the network nodes, only 18% was established. This indicates lower coherence between the network nodes. Table 3 shows the degree centrality, closeness centrality, and betweenness centrality of each node. As can be seen, nodes representing “low mood” and “cry” are the most prominent, with “social withdrawal,” “irritability,” and “shiver” following closely behind, underscoring their central importance in the network.

**Figure 3:**
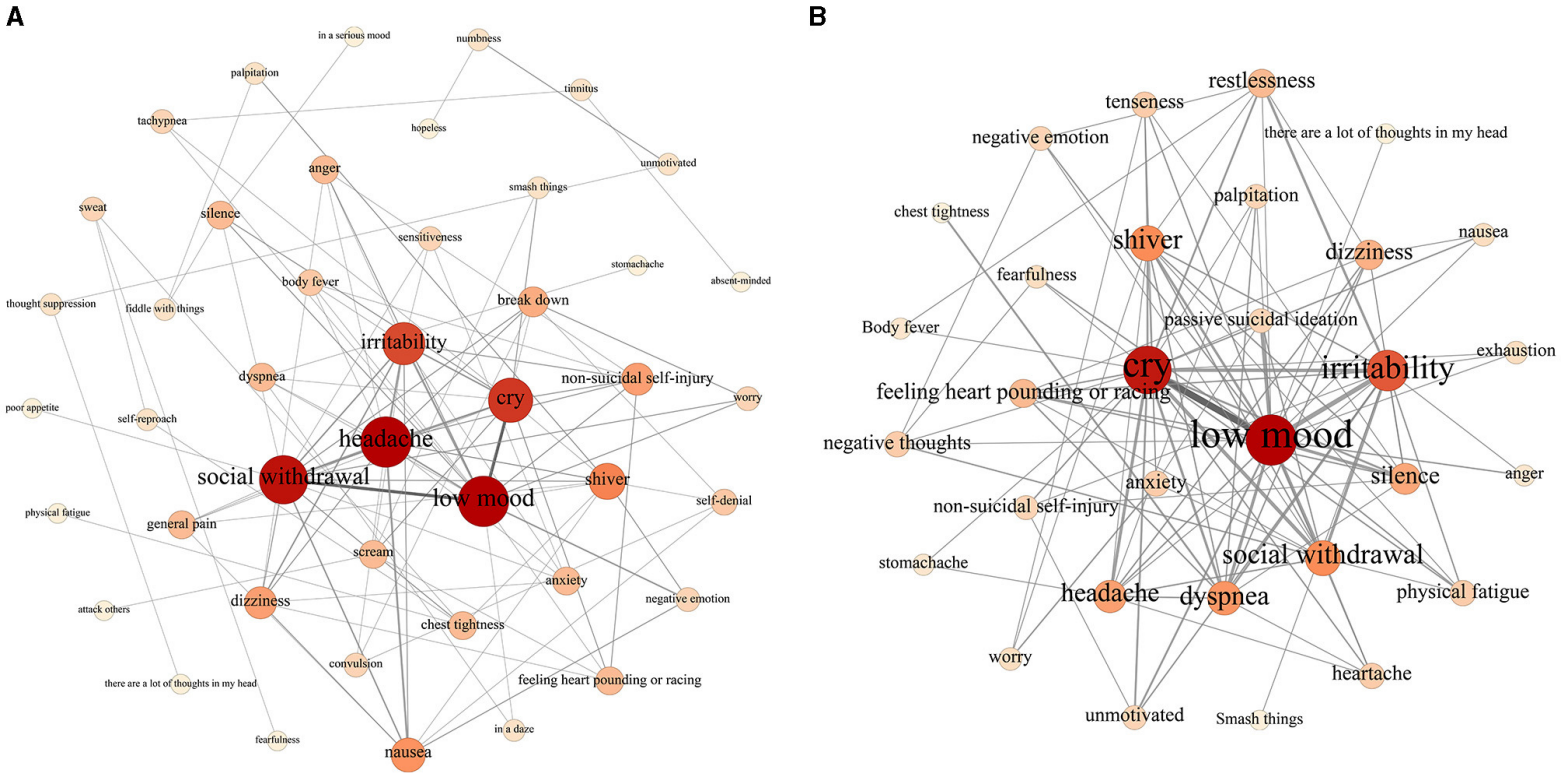
**(A)** Network structure visualization of male warning signs. **(B)** Network structure visualization of female warning signs.

Furthermore, male network (Figure 3A) consisted of 44 nodes and 101 edges, with a network density of 0.11. In contrast, the female network (Figure 3B) comprised 31 nodes and 202 edges, resulting in a higher network density of 0.21. This comparison highlights that while the male network had a greater number of nodes, it exhibited a lower edge count and density compared to the female network. This indicates that men experience a broader range of warning signs before a suicide crisis, but these signs are less interconnected. In contrast, the warning signs in the female network are more tightly connected. Regarding node degree centrality, both male and female networks prominently featured nodes such as “low mood,” “cry,” “social withdrawal,” and “irritability,” which were consistently influential across the entire network.

### 3.3 Content analysis

Directed content analysis revealed that within the sample, approximately two-thirds (76.6%, *n* = 95) of participants reported experiencing at least one emotional warning sign. Similarly, a comparable proportion reported at least one behavioral (50.8%, *n* = 63) or physiological warning sign (52.4%, *n* = 65), while 39.5% (*n* = 49) reported at least one cognitive warning sign. The chi-square test results presented in Table 4 indicated that there were no statistically significant differences in the proportion of reported warning signs among patients of different genders, ages, or histories of suicide attempts (*P* > 0.05).

**Table 4:** Comparison of warning signs depending on gender, age, and past suicidal attempts (*n* = 124).

	***n* (%)**	**Gender**			**Age**			**Past suicidal attempts**		
		**Male (*****n*** = **35)** ***n*** **(%)**	**Female (*****n*** = **89)** ***n*** **(%)**	**p-value**	**Adolescent (*****n*** = **45)** ***n*** **(%)**	**Young adult (*****n*** = **79)** ***n*** **(%)**	**p-value**	**Yes (*****n*** = **59)** ***n*** **(%)**	**No (*****n*** = **65)** ***n*** **(%)**	**p-value**
**Cognitive**				0.735			0.397			0.113
Yes	49 (39.5)	13 (37.1)	36 (40.4)		20 (44.4)	29 (36.7)		19 (32.2)	30 (46.2)	
No	75 (60.5)	22 (62.9)	53 (59.6)		25 (55.6)	50 (63.3)		40 (67.8)	35 (53.8)	
**Emotional**				0.576			0.501			0.235
Yes	95 (76.6)	28 (80.0)	67 (75.3)		36 (80.0)	59 (74.7)		48 (81.4)	47 (72.3)	
No	29 (23.4)	7 (20.0)	22 (24.7)		9 (20.0)	20 (25.3)		11 (18.6)	18 (27.7)	
**Behavioral**				0.376			0.425			0.713
Yes	63 (50.8)	20 (57.1)	43 (48.3)		25 (55.6)	38 (48.1)		31 (52.5)	32 (49.2)	
No	61 (49.2)	15 (42.9)	46 (51.7)		20 (44.4)	41 (51.9)		28 (47.5)	33 (50.8)	
**Physiological**				0.144			0.367			0.738
Yes	65 (52.4)	22 (62.9)	43 (48.3)		26 (57.8)	39 (49.4)		30 (50.8)	35 (53.8)	
No	59 (47.6)	13 (37.1)	46 (51.7)		19 (42.2)	40 (50.6)		29 (49.2)	30 (46.2)	
**Number of themes reported**				0.024			0.356			0.780
One	24 (19.4)	4 (11.4)	20 (22.5)		6 (13.3)	18 (22.8)		13 (22.0)	11 (16.9)	
Two	60 (48.4)	15 (42.9)	45 (50.6)		21 (46.7)	39 (49.4)		26 (43.3)	34 (52.3)	
Three	31 (25.0)	15 (42.9)	16 (18.0)		13 (28.9)	18 (22.8)		16 (27.1)	15 (23.1)	
Four	9 (7.26)	1 (2.9)	8 (9.0)		5 (11.1)	4 (5.1)		4 (6.8)	5 (7.7)	

Regarding the quantity of reported warning sign themes, participants most frequently reported experiencing two themes simultaneously (48.4%), followed by three themes (25.0%), one theme (19.4%), and four themes (7.3%). The chi-square test results presented in Table 4 revealed a higher proportion of males reporting three sign themes simultaneously compared to females (*P* < 0.05). However, there were no statistically significant differences in the proportions of sign themes reported among patients of varying ages or histories of suicide attempts (*P* > 0.05).

## 4 Discussion

This study extensively investigated suicide warning signs among Chinese mood disorder patients. To our knowledge, it is the first to utilize network analysis in studying suicide warning signs. Additionally, we employed content analysis to categorize these signs according to suicide mode, thereby facilitating a deeper understanding of their meaning and characteristics. These insights offer valuable contributions to suicide prevention and intervention strategies.

The majority of patients in this study reported at least one emotional warning sign (e.g., low mood, cry, anger), with over half reporting at least one physiological warning sign (e.g., headache, dizziness, insomnia) or behavioral warning sign (e.g., social withdrawal, engagement in non-suicidal self-harm), and fewer reporting at least one cognitive warning sign (e.g., feeling abandoned, rumination). Additionally, patients typically reported experiencing two to three warning signs simultaneously, primarily concentrated in emotional, behavioral, and physiological domains, suggesting that suicide risk management measures for mood disorder patients should be multifaceted.

Firstly, from the perspective of word frequency analysis and network analysis results, “low mood” and “cry” were evidently the most prominent signs, positioned at the center of the network diagram, indicating their high correlation with the remaining signs and their potential to maintain or trigger them within this sample. This pattern aligns with the tenets of the fluid vulnerability theory (33), which suggests that external events contribute to triggering or activating the suicide mode, with suicidal intent and emotional distress being active direct manifestations. Similarly, Shneidman's (36) theory of suicide describes psychache (i.e., emotional or psychological pain) as the primary motivator of a suicide attempt (37). Therefore, targeting these negative emotions and teaching patients emotion regulation and distress tolerance skills emerged as important goals for reducing suicidal ideation. Indeed, evidence has shown that treatments such as dialectical behavior therapy (38) and mindfulness training (39) significantly lowered suicide rates among individuals with emotional dysregulation.

Secondly, “social withdrawal” emerges as another crucial warning sign, closely associated with “low mood”. Previous studies have indicated a positive correlation between social withdrawal and suicidal ideation, with this correlation is influenced by emotional symptoms (40). Social withdrawal diminishes the social support available to patients, potentially exacerbating the severity of emotional symptoms, which, in turn, further intensifies social withdrawal (41, 42). This creates a vicious cycle wherein worsening emotional symptoms significantly heighten the risk of suicidal ideation (43). Consequently, clinicians must recognize that social withdrawal may elevate the risk of subsequent suicide in mood disorder patients. Social support interventions aimed to prevent suicide by providing social support and strengthening social ties may be necessary for this population (44).

Furthermore, physical symptoms such as “insomnia,” “headaches,” and “trembling” emerge as common warning signs and represent significant symptom manifestations in mood disorder patients (45). In individual patients, physical pain and depression may arise from various factors in diverse ways (19). It is crucial to recognize that pain and depression are intricately intertwined, exacerbating both physical and psychological symptoms (46), and consequently elevating an individual's suicide risk. Additionally, our findings indicate that while patients often report experiencing “passive suicidal ideation”, they seldom verbalize explicit suicidal thoughts or engage in preparatory actions. This underscores the importance for doctors or family members to closely observe patients' words and actions, identifying potential signs of suicide risk in a timely manner. Of particular note is that the combination of multiple manifestations or rapid changes in depressed mood increases the risk of suicide (47), so doctors and family members should pay attention to multiple signs exhibited by patients, including social withdrawal, engaging in self-harm behaviors, and some obvious physical symptoms, rather than solely focusing on low mood as a single indicator.

While all patients in our study reported at least one warning sign, we did not observe differences in the frequency of reported warning signs among various demographic groups within the sample, including age, gender, and history of suicide attempts. This finding aligns with Bauder's study of 88 firearm owners (14). However, it is crucial to recognize that warning signs may not be uniform across different populations, given the unique characteristics of each group. For example, adolescent females might be more influenced by hormonal changes, have poorer emotional regulation, and be more prone to negative emotions such as anxiety and depression (48). Furthermore, our study found that the male warning sign network contained more signs than the female network, though with less interconnection between these signs. It indicates that interventions for men should address multiple warning signs through comprehensive evaluations and individualized treatment plans. In contrast, the higher interconnectedness of warning signs in the female network suggests that addressing key symptoms may have a cascading effect, potentially mitigating other related symptoms. By understanding the unique network structures of warning signs in males and females, clinicians can develop more effective, personalized interventions that address the specific needs of each gender. Future research should continue to explore these differences to further develop and enhance preventive strategies. Additionally, research indicates that adolescents might prioritize emotional states such as sadness and anger more than young adults (23). It's possible that our study's broad coverage of warning signs and relatively small sample size limited our ability to detect nuanced differences. Future research endeavors could delve into more specific and detailed examinations to enhance our understanding in this area.

This study had several limitations. Firstly, the study participants are all mood disorder patients, potentially biasing results toward this specific group, including higher rates of emotional warning signs. Hence, future research should explore more diverse populations in China to validate and broaden these findings. Secondly, Data collection relies on collaboration between patients and researchers, potential researcher bias and observer effects may have influenced the generation of safety plan warning signs. Despite that we have taken several measures to mitigate these influences, the presence of such biases could still present. Lastly, categorizing certain warning signs presented difficulties. For instance, “loneliness” is a multifaceted phenomenon associated with feelings of isolation and concurrent emotional distress (49). This complexity raises questions about whether “loneliness” should be classified as “cognitive” or “emotional”. Future research could integrate Exploratory Factor Analysis or Principal Component Analysis with larger sample sizes to categorize warning signs more systematically and objectively, potentially uncovering new insights into warning sign types and group differences. Addressing these limitations in future research endeavors is essential to enhance the reliability and validity of study findings.

In conclusion, this study offers a nuanced understanding of self-identified warning signs among patients with mood disorders. Our analysis highlights the prevalence of emotional indicators, notably low mood and crying, alongside the significance of social withdrawal and physiological symptoms like insomnia. These findings underscore the necessity for comprehensive suicide risk management strategies, emphasizing interventions targeting emotional regulation and social support. While our study did not find significant differences in warning signs based on key demographic characteristics, further research is warranted to explore nuanced differences. Overall, our findings contribute to the growing body of knowledge on suicide prevention in China, emphasizing the importance of tailored interventions informed by qualitative analysis.

## Data availability statement

The original contributions presented in the study are included in the article/Supplementary material, further inquiries can be directed to the corresponding author at niu_lu@csu.edu.cn upon reasonable request.

## Ethics statement

The studies involving humans were approved by Ethics Committee of Xiangya School of Public Health, Central South University (IRB no.XYGW-2021-73; XYGW-2022-39), the Affiliated Brain Hospital of Guangzhou Medical University (2021-089), and Tianjin Anding Hospital (2023-01 and 2023-02). The studies were conducted in accordance with the local legislation and institutional requirements. Written informed consent for participation in this study was provided by the participants' legal guardians/next of kin.

## Author contributions

HZ: Conceptualization, Data curation, Formal analysis, Investigation, Methodology, Writing – original draft. JZ: Conceptualization, Data curation, Formal analysis, Investigation, Writing – review & editing. LN: Conceptualization, Data curation, Funding acquisition, Methodology, Project administration, Supervision, Validation, Writing – review & editing. MZ: Data curation, Investigation, Writing – review & editing. TC: Data curation, Investigation, Writing – review & editing. YC: Data curation, Investigation, Writing – review & editing. XH: Resources, Supervision, Writing – review & editing. HT: Resources, Supervision, Writing – review & editing. YM: Resources, Supervision, Writing – review & editing. KL: Resources, Supervision, Writing – review & editing. LZ: Project administration, Resources, Supervision, Writing – review & editing.

## Footnotes

^1^Zhu, J., Niu, L., Hou, X., Tao, H., Ma, Y., Silenzio, V., et al. Feasibility and acceptability of ecological momentary assessment to assess suicide risk among young people with mood disorder: A mixed-methods study in Chinese culture. (2023).
