# Supplementary material for: Suicide warning signs of self-identification in patients with mood disorders: a qualitative analysis based on safety planning
Source: Front Public Health. 2024 Aug 21;12:1417817. doi: 10.3389/fpubh.2024.1417817 (PMC11372480; doi:10.3389/fpubh.2024.1417817)
Supplement: Supplementary file 1 [file Table_1.DOCX]

| **Table1**  Coding system for content analysis of warning signs | | | | |
| --- | --- | --- | --- | --- |
| Category | 编码 | | Code | |
| 1.Cognitive | | 1a.消极想法（如被动死亡念头） | | 1a. Negative thoughts (e.g. passive suicidal ideation)^5^ |
|  |  | 1b.绝望 | | 1b. Hopelessness^1,2,3^ |
|  |  | 1c.认知：自我、他人、未来 | | 1c. Cognitive Triad: beliefs about the self, others, the future^1^ |
|  |  | 1d.补偿策略(过度补偿，完美主义，人际关系中的屈从) | | 1d. Compensatory strategies：Overcompensation, perfectionism, subjugation in relationships^1^ |
|  |  | 1e.认知障碍(反刍、认知僵化、思维抑制、信息处理能力受损) | | 1e. Cognitive disturbance (ruminations, rigidity, thought suppression, ruminative flooding, impaired ability to process information)^2^ |
|  |  | 1f.条件规则/假设 | | 1f. Conditional rules/assumptions^1^ |
|  |  | 1g.幻觉 | | 1g. Hallucination^5^ |
| 2.Emotional | | 2a.暴怒、愤怒、敌意、寻求报复 | | 2a. Rage, anger, hostility, seeking revenge^2,3^ |
|  |  | 2b.焦虑/紧张/恐惧 | | 2b. Anxiety/ tension /fear^3^ |
|  |  | 2c.情绪痛苦（悲伤、空虚、受伤） | | 2c. Emotional pain (sadness, emptiness, hurt)^3^ |
|  |  | 2d.极大的内疚和羞愧 | | 2d. Great guilt or shame^4^ |
|  |  | 2e.孤独 | | 2e. Loneliness^1^ |
|  |  | 2f.情感耗损/衰竭/麻木 | | 2f. Emotional depletion/exhaustion/numbness^3^ |
|  | | 2g.急性快感缺乏 | | 2g. Acute anhedonia^3^ |
| 3.Behavioral | | 3a.谈论自杀 | | 3a. Talk about suicide^3^ |
|  |  | 3b.为自杀做准备（如写遗嘱或事务安排） | | 3b. Making preparations of personal affairs to attempt suicide^1,2,3,4^ |
|  |  | 3c.向特定的人传达痛苦或试图这样做 | | 3c. Communicating distress to a specific person or attempting to do so^3^ |
|  |  | 3d.酒精/药物滥用 | | 3d. Using drugs or alcohol more often^2,3,4^ |
|  |  | 3e.回避社交 | | 3e. Social withdrawal^2,3,4^ |
|  |  | 3f.鲁莽行事或从事冒险活动 | | 3f. Acting reckless or engaging in risky activities^2,3,4^ |
| 4.Physiological | | 4a.过度唤醒（易激惹、过度警觉、烦躁、失眠） | | 4a. Hyperarousal (Agitation, Hypervigilance, Irritability, Insomnia)^1,3,4^ |
|  | | 4c.躯体症状(头部、胃部和胸部等问题) | | 4c. Somatic symptoms（Head, stomach, and chest issues）^1,3,4^ |

**Supplementary file**

^1^ System Characteristicsfor the Suicidal Mode(1)

^2^ List of warning signs by a panel of experts(2)

^3^Support from literature on other warning sign studies(3,4)

^4^ Warning signs listed by the National Institute of Mental Health

^5^Added inductively based on a qualitative review of transcripts.

**Reference**

1. Rudd MD. The suicidal mode: a cognitive-behavioral model of suicidality. *Suicide Life Threat Behav* (2000) 30:18–33.

2. Rudd MD, Berman AL, Joiner TE, Nock MK, Silverman MM, Mandrusiak M, Van Orden K, Witte T. Warning Signs for Suicide: Theory, Research, and Clinical Applications. *Suicide Life Threat Behav* (2006) 36:255–262. doi: 10.1521/suli.2006.36.3.255

3. Conner KR, Kearns JC, Denneson LM. Qualitative analysis of hospital patient narratives of warning signs on the day of their suicide attempt. *Gen Hosp Psychiatry* (2022) 79:146–151. doi: 10.1016/j.genhosppsych.2022.11.001

4. Bauder CR, Hay JM, McClung JG, Starkey AG, Bryan CJ. Content Analysis of Warning Signs Identified as Part of Crisis Response Planning in a Community Sample of Gun Owners and Non-owners. *Front Psychiatry* (2022) 13:867332. doi: 10.3389/fpsyt.2022.867332

5. National Institute of Mental Health (NIMH). 2023. Warning Signs of Suicide. [online] Available at: <https://www.nimh.nih.gov/health/publications/warning-signs-of-suicide>
